# Bone Turnover Markers and Bone Mineral Density in Children with Hypophosphatemic Rickets

**DOI:** 10.3390/jcm11154622

**Published:** 2022-08-08

**Authors:** Izabela Michałus, Anna Łupińska, Izabela Woch, Katarzyna Wieczorek-Szukała, Danuta Chlebna-Sokół, Andrzej Lewiński

**Affiliations:** 1 Department of Endocrinology and Metabolic Diseases, Polish Mother’s Memorial Hospital-Research Institute, 93-338 Lodz, Poland; 2 Department of Paediatric Endocrinology, Medical University of Lodz, 90-419 Lodz, Poland; 3 Department of Paediatrics, Neonatal Pathology and Metabolic Bone Diseases, Medical University of Lodz, 91-738 Lodz, Poland; 4 Department of Endocrinology and Metabolic Diseases, Medical University of Lodz, 90-419 Lodz, Poland

**Keywords:** hypophosphatemic rickets, bone turnover, bone mineral density

## Abstract

Hypophosphatemic rickets is a rare disease that results in bone deformities. However, little is known about bone turnover and bone mass disorders in this disease. This retrospective study included 12 children aged 1–16 years diagnosed with hypophosphatemic rickets. Parameters of calcium-phosphate metabolism and bone turnover markers were analysed. Bone mineral density was assessed with the use of dual-energy X-ray absorptiometry, and indices of quantitative ultrasound examination of tibiae and radial bones were analysed. In the majority of patients, hypophosphatemia and hyperphosphaturia were present. The assessed bone turnover markers showed increased bone formation. Increased pyridinoline levels were found in 5 out of 12 patients. Bone mineral density was decreased only in one patient. Decreased values of quantitative ultrasound examination were observed in all the analysed patients. Conclusions: (1) Bone metabolism disturbances, reflected in the increased values of bone turnover markers and worse bone quality, were found in the group of patients with hypophosphatemic rickets. (2) It is crucial to determine bone turnover markers, dual-energy X-ray absorptiometry findings and indices of quantitative ultrasound examination in order to monitor progress of the disease, as well as treatment effects.

## 1. Introduction

Among all cases of rare genetically conditioned rickets, hypophosphatemic rickets is diagnosed most commonly; its incidence is estimated at 1 in 20,000 births [1,2]. At present, the term “hypophosphatemic rickets” covers a group of disorders with a similar phenotype but with different genotypes, inheritance models and etiopathogenesis. Mutations of 10 or even more genes underlying this disease have been described so far [2,3,4]. The studies on the pathogenesis of hypophosphatemic rickets made it possible to distinguish FGF-23-dependent and non-FGF-23-dependent hypophosphatemic rickets [2,5]. 

**Figure 1:**
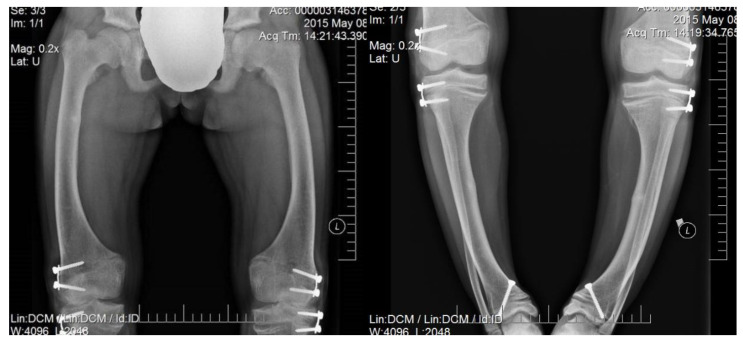
A 15-year-old girl diagnosed with hypophosphatemic rickets; significant varus deformity of the lower extremities is visible in spite of surgical and medical treatment.

**Figure 2:**
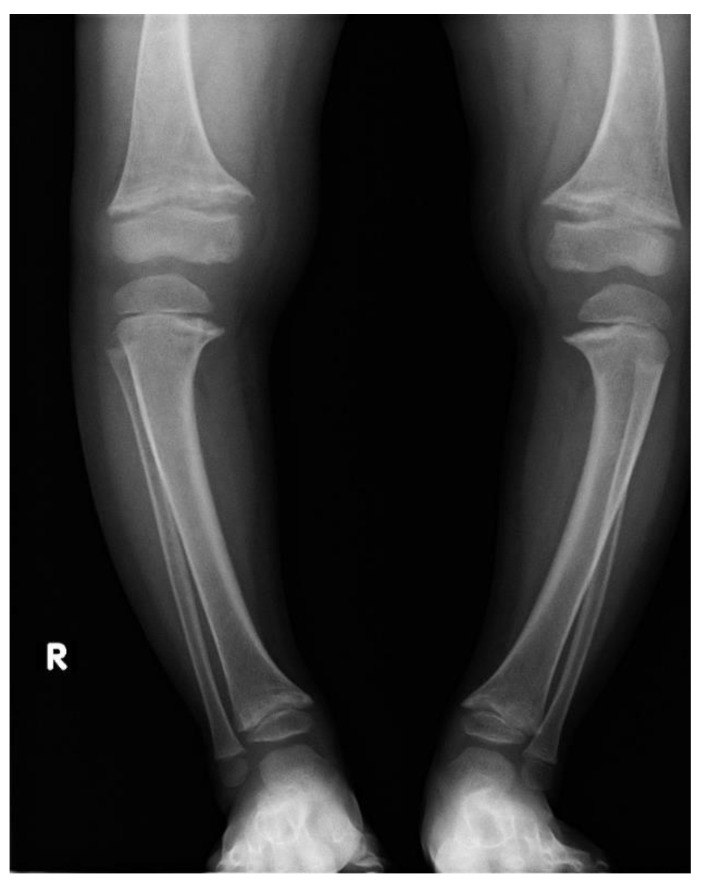
A 5-year-old girl diagnosed with hypophosphatemic rickets; significant varus deformity of the lower extremities.

Irrespectively of inheritance pattern, all congenital forms of hypophosphatemic rickets present similar phenotypes including short height along with age and typical changes in the skeleton. Characteristic bone symptoms of hypophosphatemic rickets include varus deformity of the lower extremities, frequently severe, which appears after the child starts walking—usually in the 2nd year of life—that also progresses with age and may lead to gait disturbances and require surgical correction [6,7] (Figure 1 and Figure 2). 

Other symptoms present also in different types of rickets include the following: short stature (non-treated adults reach body height of 130–165 cm), weakening of muscle strength and exercise performance, expansion of long bone metaphyses, rachitic bracelets and rachitic rosary. Skull bone deformities may include craniosynostosis, protruding forehead and hypoplastic central part of the face, as well as peridental lesions and elongation of teeth crowns. Adults in whom the disease was not diagnosed and no treatment was implemented develop multiarticular pains, chronic fatigue, depression, symptoms suggestive of ankylosing spondylitis and entezopathies (calcification of the tendons, ligaments and articular capsules) [5,7,8,9,10]. 

Laboratory tests show significant hypophosphatemia with hyperphosphaturia that is caused by decreased phosphate reabsorption in kidney tubules. Increased phosphaturia in hypophosphatemic rickets is accompanied by a decrease in the tubular reabsorption of phosphate (TRP) below 85%.

Calcium levels remain in normal ranges or are slightly decreased. Alkaline phosphatase (ALP) activity is elevated in hypophosphatemic rickets, and a decrease in its activity is a good marker of treatment efficacy. The bone formation marker osteocalcin and the bone resorption marker pyridinoline may be elevated, which reflects accelerated bone remodelling [5,7,11]. 

The levels of the hepatic vitamin D metabolite (25OHD) remain normal as long as cutaneous synthesis is maintained or the patient receives relevant vitamin D supplementation in preventive doses, the same as in the healthy population. The levels of the renal metabolite may be decreased in FGF-23-dependent rickets or increased in FGF-23-independent rickets [7,11]. 

Treatment depends on the form of hypophosphatemic rickets and on clinical disease manifestation. In the FGF-23-dependent form, in children, treatment starts with the administration of active vitamin D analogues such as alfacalcidol (at a dose of 1–3 ug once daily) or calcitriol. Additionally, if there is vitamin D deficiency, cholecalciferol supplementation should be started in accordance with typical guidelines for a given population. This should be the sequence of treatment: vitamin D products before the treatment with phosphates prevents occurrence or exacerbation of preexisting secondary hyperparathyroidism, hypercalciuria and consequent nephrocalcinosis. After one week of treatment with vitamin D, phosphates are started at a dose of 40–70 mg/kg/day in 3 to 8 doses, optimally without a night-time break. The effect of conventional treatment in growth has been related to the age at diagnosis and onset of treatment, and some patients do not respond to this treatment. 

In recent years, burosumab, a recombinant human IgG1 monoclonal antibody, has been used for treatment. Among patients treated with the recombinant human IgG1 monoclonal antibody, significant improvements in serum phosphate levels and a reduction in pain alongside clinical improvement of patients have been demonstrated.

As low stature is one of the leading symptoms of hypophosphatemic rickets, it is growth hormone (GH) that has so far most often been considered an alternative treatment option for hypophosphatemic rickets. GH treatment can increase short-term linear growth in these patients before puberty, but clinical observations have shown that it has been ineffective in some patients and has caused worsening in limb deformity, radiographic deterioration and biochemical abnormalities. 

When pharmacological treatment is ineffective in patients with significant bone deformities, surgical procedures such as corrective osteotomy, epiphysiodesis or epiphyseal cartilage block of the knee and ankle joints using Blount staplers are used. Corrective osteotomies are rarely performed in children under 6 years of age, as pharmacological treatment very often improves lower limb deformities in younger children. This procedure is usually postponed until the growth process is complete. Severe deformities may require earlier treatment, and then a newer, less invasive method is used, epiphysiodesis, which induces corrective, differential growth of the growth plate [4,6,7,12].

The aim of this study has been to analyse bone turnover markers and bone mineral density in children with hypophosphatemic rickets. So far, there are only a few reports in the literature on the assessment of bone mineralisation and bone turnover markers in children with hypophosphatemic rickets.

From a clinical point of view, in patients with metabolic bone disease, and therefore also hypophosphatemic rickets, it is important to assess bone quality in terms of potential fracture risk. Therefore, the authors analysed the results of the gold standard for assessing bone mineral density, DXA, and the less commonly performed non-invasive ultrasound QUS examination. The analysis will select appropriate diagnostic methods to assess bone quality and thus the potential risk of fractures.

## 2. Material and Methods

Biochemical parameters of bone turnover, such as alkaline phosphatase, osteocalcin and pyridinoline, were assessed in all the patients. Calcium and phosphate levels were measured in serum, in 24-h urine collection and/or in the first morning urine portion. Serum parathormone activity (PTH) as well as both renal and hepatic vitamin D metabolite levels were measured. Furthermore, TRP was calculated according the following formula: TRP% = 100 − [1 − (P in urine × creatinine in serum)/(P in serum × creatinine in urine)]). 

Serum concentrations of calcium, phosphate levels and ALP activity were determined using routine analytical methods. Serum values of 25-OHD and PTH were determined using electrochemiluminescence detection (Architect, Abbott, IL, USA), as was osteocalcin (Cobas 8000 [COBAS e602] Roche, Basel, Switzerland). Pyridinoline was assayed with chemiluminescence (Immulite 2000 XPi, Siemens, Erlangen, Germany). Bone mineral density was assessed with dual-beam X-ray absorptiometry (DXA) using the same scanner (GE Lunar Prodigy) in total body and spine mode. Z-score was calculated for all the patients. Quantitative ultrasound examination (QUS) was assessed with a Sunlight Omnisense 7000P apparatus. The speeds of the ultrasound waves (speed of sound; SOS) along the tibiae and radial bones were measured. X-ray examination of lower extremities was also performed in all patients. The influence of hypophosphatemic rickets on skeletal mass is not good, especially for children, although studies report variable results. As reported in literature, most untreated adults have normal indices of bone mass. Studies using dual-energy X-ray absorptiometry (DXA) in these patients also suggest that bone mineral density (BMD) is increased at the spine but not at cortical sites. Quantitative ultrasound (QUS) is a non-invasive and radiation-free technology that is used for bone mass and quality assessment. Studies in adults confirm that the information provided by QUS measurements is independent of BMD because it only relates to cortical bone [13].

### Study Population

This assessment included 12 patients aged 1–16 years (6 girls and 6 boys) treated in the Department of Paediatric Propedeutics and Bone Metabolic Diseases and in the Outpatient Clinic of Osteporosis and Other Bone Metabolism Diseases. The patients were diagnosed with hypophosphatemic rickets based on clinical symptoms and biochemical disease markers; in eight children, the diagnosis was confirmed by genetic testing. The study was retrospective.

## 3. Results

Table 1 presents the anthropometric characteristics and pubertal stages of the patients during first hospitalization, mainly for diagnosing. Six boys and six girls were assessed. Nine of the twelve children were in the pre-pubertal stage at the time of the study. Body height deficit was found in 7 out of 12 children, while only 2 of the assessed patients had body mass deficits.

Medical history and clinical analysis of the examined patients showed that various deformities of the lower limbs were the main symptoms in all the patients and that it was the reason for the patients’ referrals for laboratory tests. Additionally, 4 out of 12 children had gait disturbances, the same number of patients suffered from bone and joint pain, and 1 out of these 12 children had experienced a finger fracture. Family history was positive in 5 children. All patients were treated with phosphates, and three of them underwent corrective surgery (Table 2).

The analysis of calcium–phosphate metabolism results showed that hypophosphatemia was present in the majority of patients (10 of 12) and documented in consecutive measurements; serum calcium levels were normal in all patients. Hyperphosphaturia occurred in the majority of children from the analysed group (10 of 12). Increased urine phosphate excretion was recorded both in the 24-h urine collection and in the calculated phosphate elimination ratio in the first morning urine portion. Both hypophosphatemia and hyperphosphaturia are typical findings for the disease in question. In our study the aforementioned biochemical changes were the first stimulus for the further diagnosis of hypophosphatemic rickets. Calculated phosphate reabsorption in the renal tubules was decreased in 8 out of 12 patients. The levels of the renal vitamin D metabolite were normal in all 12 children, whereas those of the hepatic metabolite were decreased in as many as 7 of 12 of the subjects.

The assessed bone turnover markers showed, in all patients, increased bone formation markers. Accordingly, values of ALP and osteocalcin were found elevated in 10 of 12 and 7 of 12 patients, respectively. The levels of pyridinoline, a bone resorption marker, were higher than the upper limit of reference values in 5 of 12 patients. Only in two patients (P1 and P10) did both bone formation and bone resorption markers remain normal, whereas in 5 of 12 children, increased values of at least one bone formation marker and one resorption marker were observed. The results are presented in Table 3.

In 9 of 12 patients, the bone mineral density was assessed by densitometry. In the total body projection, bone density was decreased only in one girl with the most pronounced short stature, in whom the correct diagnosis and treatment had been delayed. On the other hand, we observed an elevated Z-score in the spine Z-score programme (>2.0) in one (1) child. In all analysed patients, decreased Z-scores were present on quantitative ultrasound bone examination at at least one measurement site within the tibiae and/or radial bones (Table 4).

## 4. Discussion

Hypophosphatemic rickets is a rare disease with a diverse genetic background. Diagnosis is based on clinical symptoms in combination with imaging and laboratory results. In addition to measurements of basic calcium–phosphate metabolism parameters, where significant hyperphosphaturia accompanied by hypophosphatemia predominate, the assessment of bone turnover markers (both bone formation markers and bone resorption markers), e.g., ALP, osteocalcin and pyridinoline, is indicated. Increased activity of these markers reflects increased bone metabolic activity that even in the absence of fractures may cause exacerbation of bone deformities, e.g., varus deformity of the lower extremities. So far, there are only a few studies that analyse bone turnover and bone mineralisation in children with hypophosphatemic rickets [14,15,16].

In our study, the analysis of the calcium-phosphate balance results showed that the majority of patients had hypophosphatemia, but serum calcium levels were normal in all patients. Hyperphosphaturia was present in the majority of children in the analysed group. Calculated renal tubular phosphate reabsorption was reduced in 8 of 12 patients. The bone turnover markers assessed showed mainly increased activity of bone formation markers. Bone resorption marker levels were higher than the upper limit of reference values in about half of the patients. In only two did both bone formation and bone resorption markers remain normal. The assessment of bone mineral density using densitometry, in 9 of 12 patients, showed that bone mineral density was normal. In the total body projection, bone density was reduced in only one girl in whom the correct diagnosis and treatment had been delayed. All analysed patients had reduced Z-scores on the quantitative bone ultrasound examination.

Bone turnover markers in the assessment of hypophosphatemic rickets were analysed by Nagata et al. [14]. The authors showed elevated levels of the procollagen type 1 N-terminal propeptide (PINP) in a group of patients with hypophosphatemic rickets compared with the control group, which suggested bone formation stimulation [14]. Similarly, bone formation processes predominated in our patients, which was manifested by the increased values of ALP and/or osteocalcin in 10 out of 12 assessed children.

Shanbhogue et al. [15] conducted a 6-year analysis of bone mineral density and bone turnover markers in adult patients diagnosed with hypophosphatemic rickets, in groups receiving and not receiving conventional treatment. In this study, no effect of the treatment on bone mineral density at the adult age was found. It corresponds with the recommendation that the treatment be applied in adults only if the disease symptoms are present or if the patient is prepared for surgical intervention [6,7]. The previous quoted study showed that the continuation of the conventional treatment in adults was safe and evoked no significant changes in bone mineral density (BMD) [15]. It was also determined that in patients who started the treatment in childhood, the disease took a milder course in comparison with the untreated patients.

Due to the clinical manifestation of the disease that hypophosphatemic rickets predominantly affects the motor system, the assessment of bone mineral density seems important, and the BMD may be decreased or increased in densitometric measurements.

Colares et al. [16] reported that DXA examination results should be assessed with caution in those children with hypophosphatemic rickets in whom the consequences of anatomy-related and anthropometric factors were observed. Additionally, HR-pQCT analysis suggests that hypophosphatemic rickets mainly affects trabecular bone metabolism, to a larger extent in the tibiae than in the radial bone, and that appropriate treatment positively affects bone mineralisation, mainly of the cortical part.

Cheung et al. [17] assessed volumetric bone mineral density (vBMD) with quantitative computed tomography of the forearm. They showed that patients with PHEX mutations who received calcitriol and phosphate supplementation had elevated trabecular vBMD in the distal segment of the radial bone but low vBMD in the diaphyseal cortex of the radial bone. Decreased vBMD in the cortical layer probably reflects an underlying mineralisation defect that is not fully compensated by current treatment methods. Adult patients who do not continue the treatment have decreased volumes both of the trabecular bone and of the cortical layer. In general, vBMD of the cortical bone was low in all age groups and in all treated groups, but Z-score was relatively higher in children currently under treatment than in the groups of adult patients who discontinued the treatment or were never treated.

The above reports show that a normal DXA result does not guarantee that the patient has normal BMD and is appropriately treated. Quantitative CT is a better method for this assessment.

The QUS technique that we have used for the examination of our patients is a simple and non-invasive method which does not expose the patient to radiation. It was found that the measurement of sound velocity (SOS) along a long bone gave information not only about bone density but also about its’ microarchitecture, cortical thickness and elasticity [18,19,20]. Decreased Z-score in the quantitative ultrasound examination of the tibiae and radial bones in the majority of our patients may be caused by the fact that the cortical layer is the most affected by mineralisation disturbances, which reflects the poorer quality of these bones and may result in fracture occurrence in the future.

Taking all into account, in children with hypophosphatemic rickets, typical biochemical disturbances such as chronic hyperphosphaturia and hypophosphatemia may be accompanied by accelerated bone turnover; therefore, monitoring of bone formation and bone resorption markers is also recommended in this disease. Disturbances of mineral metabolism in hypophosphatemic rickets may manifest in the form of either increased or decreased BMD. The decreases in the indices of QUS in all examined patients suggest poorer bone cortex quality in this disease.

## 5. Conclusions

In the evaluated group of children with hypophosphatemic rickets, bone metabolism abnormalities were found manifested in elevated bone turnover markers and poorer bone quality. These parameters need to be monitored during the treatment process.

In children with hypophosphatemic rickets, the assessment of bone status cannot be based solely on the result of a densitometric examination because a normal result does not indicate correct bone quality. An important complementary role is played by bone ultrasound examination, which is non-invasive but rarely performed and which, by assessing cortical bone, gives better information on bone quality. Abnormal bone quality can predispose to fractures, just as in patients with normal bone mineral density.

## Figures and Tables

**Table 1:** Anthropometric characteristics of the examined patients diagnosed with hypophosphatemic rickets.

Patient Number	Gender	Age (Years)	High (cm/Percentyl)	Weight (kg/Percentyl)	Tanner Stage
P1	F	14	127.6/<3	29/<3	I/II
P2	F	5	107/10–25	23/90–97	I
P3	M	3.5	98/25	19/90	I
P4	M	1	80/25–50	13/90–97	I
P5	M	4	97.8/3–10	18.5/50–75	I
P6	M	4	96.3/<3	22/90–97	I
P7	F	11	134/<3	38.3/50–75	III
P8	F	3	92.8/10–25	17/75–90	I
P9	M	16	161/<3	59/25–52	IV
P10	F	2	78.8/<3	10/3	I
P11	F	4.5	98.2/<3	18.8/50–75	I
P12	M	14	160.5/10–25	55/50–75	III/IV

**Table 2:** Clinical characteristics of the examined patients diagnosed with hypophosphatemic rickets.

Patient	Clinical Symptoms						Medical Interview	Genetic Study/Mutation	Treatment	
	Varus Deformity of the Lower Limbs	Other bone Deformities	Gait Disturbances	Bone Pain	Fractures	Short Stature			Pharmacological	Surgery
P1	Yes	Yes (deformations of the rib arches)	Yes	No	No	Yes	Negative	Not performed	Yes	Yes
P2	Yes	Yes (lumbal hyperlordosis)	Yes	No	No	No	Negative	Not performed	Yes	Yes
P3	Yes	No	No	No	No	No	Positive -hypophosphatemic rickets/brother	Positive-PHEX gene mutation	Yes	No
P4	Yes	Yes (deformations of the rib arches)	No	No	No	No	Positive-hypophosphatemic rickets/brother	Positive-PHEX gene mutation	Yes	No
P5	Yes	No	No	Yes	No	Yes	Positive-hypophosphatemic rickets/mother	Positive-PHEX gene mutation	Yes	No
P6	Yes	No	Yes	No	No	Yes	Negative	Positive-PHEX gene mutation	Yes	No
P7	Yes	Yes (deformations of the rib arches)	Yes	Yes	No	Yes	Negative	Positive-PHEX gene mutation	Yes	Yes
P8	Yes	No	No	No	No	No	Negative	Pending	Yes	No
P9	Yes	No	No	No	Yes	Yes	Positive-hypophosphatemic rickets/mother and grandmother	Positive-PHEX gene mutation	Yes	No
P10	Yes	No	No	No	No	No	Positive-hypophosphatemic rickets/mother	Pending	Yes	No
P11	Yes	No	No	Yes	No	Yes	Negative	Positive-PHEX gene mutation	Yes	No
P12	Yes	Yes (scoliosis)	No	Yes	No	No	Negative	Positive-PHEX gene mutation	Yes	No

**Table 3:** The results of testing the calcium–phosphate metabolism and bone metabolism in patients with hypophosphatemic rickets.

Calcium–Phosphate and Bone Turnover Test *(n*)	Results		
	Decreased	Normal	Increased
Calcium (mg/dl)—serum (*n* = 12)	-	12	-
Phosphorus (mg/dl)—serum (*n* = 12)	10	2	-
Calcium (mg/kg/24 h) 24-h urine collection (*n* = 9)	-	9	-
Phosphorus (mg/kg/24 h) 24-h urine collection (*n* = 8)	-	8	4
Calcium/creatinine Ratio in the first urine portion (*n* = 12)	-	12	-
Phosphorus/creatinine Ratio in the first urine portion (*n* = 12)	-	3	9
TRP (%) (*n* = 12)	8	4	-
25(OH)D (ng/mL) (*n* = 12)	7	5	-
1,25(OH)2D (pg/mL) (*n* = 12)	-	12	-
Parathormone (pg/mL) (*n* = 12)	-	12	-
Alkaline phosphatase (U/L) (*n* = 12)	-	2	10
Osteokalcin (ng/mL) (*n* = 11)	-	4	7
Pyrlinx D (nmol/mmol) (*n* = 9)	-	4	5

**Table 4:** Bone densitometry and ultrasound examination results in patients diagnosed with hypophosphatemic rickets.

Patient	DXA				QUS							
	Total Body (TBLH)		Spine L1–L4		Right Radius		Left Radius		Right Tibia		Left Tibia	
	BMD (g/cm^2^)	Z-Score	BMD (g/cm^2^)	Z-Score	SOS m/s	Z-Score	SOS m/s	Z-Score	SOS m/s	Z-Score	SOS m/s	Z-Score
P1	0.668	−2.2	1.043	0.2	3671	−1.8	3565	−2.8	3450	−3.4	3701	−0.7
P2	-	-	0.878	2.3	3202	−5.5	3147	−6.1	3126	−5.5	3131	−5.5
P3	0.606	0.92	-	-	3313	−3.3	3586	−0.7	3301	−3.2	3549	−0.2
P4	-	-	-	-	3367	−2.0	3041	−3.4	3193	−0.7	3153	−1.2
P5	0.554	1.0	-	-	3300	−3.7	3165	−5.0	3050	−6.5	3050	−6.5
P6	0.542	0.5	0.682	1.5	-	-	-	-	3094	−6.5	3117	−5.8
P7	-	−1.2	0.694	1.6	3579	−2.1	3584	−2.0	3432	−2.8	3480	−2.2
P8	-	-	-	-	3164	−4.4	3199	−4.0	3231	−3.1	3160	−3.9
P9	-	0.3	-	1.2	3481	−3.5	3475	−3.5	3639	−1.4	3616	−1.7
P10	-	-	-	-	3387	1.1	3433	1.6	3389	−2.4	3590	2.4
P11	0.493	−0.1	-	-	3583	−1.2	3459	−2.4	3532	−0.9	3455	−1.7
P12	-	−0.9	-	1.2	3470	−3.2	3475	−3.2	3533	−3.3	3432	−3.3

## Data Availability

The datasets used and/or analysed within the framework of this study are available from the corresponding author on reasonable request.
